# Association between circulating mononuclear cell mitochondrial DNA copy number and in-hospital mortality in septic patients: A prospective observational study based on the Sepsis-3 definition

**DOI:** 10.1371/journal.pone.0212808

**Published:** 2019-02-22

**Authors:** Yi Yang, Jingjuan Yang, Biying Yu, Li Li, Lin Luo, Fengfeng Wu, Binbin Wu

**Affiliations:** 1 Kidney Disease Center, the First Affiliated Hospital, College of Medicine, Zhejiang University, Key Laboratory of Kidney Disease Prevention and Control Technology, Zhejiang Province; The Third Grade Laboratory under the National State, Administration of Traditional Chinese Medicine, Hangzhou, China; 2 Department of Nephrology, the Fourth Affiliated Hospital, College of Medicine, Zhejiang University, Yiwu, China; University of Mississippi Medical Center, UNITED STATES

## Abstract

**Purpose:**

To explore the association between circulating mononuclear cell mitochondrial DNA copy number and the prognosis of sepsis patients based on the Third International Consensus Definitions for Sepsis and Septic Shock (Sepsis-3 definition).

**Methods:**

A total of 200 adult patients who had recently devoloped sepsis were prospectively recruited as the study cohort. Demographic and clinical data were recorded along with a 28-day outcome. Mononuclear cell mtDNA copy number was assessed by quantitative PCR.

**Results:**

The 28-day outcome of sepsis patients was significantly associated with circulating mononuclear cell mtDNA copy number. The median mononuclear cell relative mtDNA copy number of survivors was significantly higher than that of nonsurvivors (406.68, range 196.65–625.35 vs. 320.57, range 175.98–437.33, p = 0.001). The Cox proportional hazard survival model analysis indicated that mononuclear cell relative mtDNA copy number was significantly negative associated with the 28-day outcome. For every additional unit of mononuclear cell mtDNA relative copy number, the risk of death falls by 0.1% (HR = 0.999, 95% CI = 0.998 to 1.000, *p* = 0.017).

**Conclusions:**

Our data indicate first that circulating mononuclear cellular mtDNA copy number might be helpful for outcome predictions in sepsis patients, and second that lower mtDNA copy number implied poor prognosis.

## Introduction

According to Global Burden of Disease 2015 Study, infection is one of the leading causes of global mortality [1]. The Third International Consensus Definitions for Sepsis and Septic Shock (Sepsis-3 definition), defined sepsis as organ dysfunction caused by a dysregulated host response to infection [2]. Based on the latest guidelines and consensus, organ dysfunction became the most important part of the definition of sepsis [3]. The update of the definition is essential for the epidemiologic studies and clinical trials, especially regarding controversial issues. Although the mechanisms involved in the pathophysiology of sepsis remain unclear, several lines of evidence suggest that mitochondrial dysfunction is implicated in its pathogenesis [4, 5]. According to the endosymbiotic theory, mitochondria evolved from ancient bacteria and had their own genomes that were similar to those of bacterial DNA [6]. During sepsis, mitochondria could generate a large amount of reactive oxygen species (ROS), leading to mitochondrial DNA (mtDNA) damage and mutation [7]. Damaged mtDNA act as a damage-associated molecular pattern (DAMP) when released into extracellular enviroment, then elicit innate immune responses and cause cascades of expanding inflammation [8, 9]. In many mitochondrial disorders, mutation and depletion of mtDNA may affect ATP synthesis, thus reducing energy metabolism and leading to organ dysfunction [10]. However, there remain controversial points regarding the association between mtDNA and outcomes of patients with sepsis.

According to the old definition of sepsis, it is difficult to discriminate the disease stages with various characteristics of the pathophysiological mechanisms. Moreover, mitochondria show dynamic changes at different stages of sepsis, in particular, with or without organ dysfunction [11]. Therefore, selecting patients based on previous sepsis definition for mtDNA copy number studies might generate controversy [12–14]. Considering the new definition of sepsis, it remains necessary to study the association between cell mtDNA copy number and prognosis of septic patients with clear organ dysfunction.

Considering these elements, the objective of the present study was to determine whether circulating mononuclear cell mtDNA copy number was associated with in-hospital mortality in patients with sepsis based on the Sepsis-3 definition. It might help reveal the role of mtDNA depletion in sepsis-related sequential organ injury.

## Materials and methods

### Study population

In accordance with the grouping definition based on mtDNA copy number (high or low), the sample size was estimated based on a priori power calculation indicating an 80% power to detect a 2-fold difference in risk for low mtDNA copy number frequency (50%) between nonsurvivors and survivors at the 0.05 significance level using a power and sample size website (http://powerandsamplesize.com). We performed a prospective, observational study in 200 adult patients who had recently developed sepsis between October 2014 and May 2016 in the intensive care unit (ICU) at the First Affiliated Hospital, College of Medicine, Zhejiang University. Patients were recruited in the present study according to the Third International Consensus Definitions for Sepsis and Septic Shock (Sepsis-3 definition): sepsis was defined as organ dysfunction caused by a dysregulated host response to infection. Organ dysfunction was represented by a Sequential Organ Failure Assessment (SOFA) score of 2 points or more. Patients were excluded if they were younger than 18 years; or were unable to give written informed consent. The hospital’s Institutional Review Committee on Human Research approved the study protocol and all of the patients provided written informed consent.

### Variables and sample collection

Demographic variables, age, gender, comorbidities, vital signs, laboratory data, origin of infection in patients with sepsis, the SOFA score at the ICU admission, and outcome were collected. We used death during the 28-day period or survival at 28 days after ICU admission as endpoints. Follow-up was completed for all study patients. Blood samples were collected upon admission to ICU. Peripheral venous blood was collected into EDTA-containing tubes. Genomic DNA was isolated and stored for mtDNA analysis. We determined mtDNA relative copy number after clinical data were obtained.

### DNA isolation from peripheral blood mononuclear cells (PBMCs)

PBMCs were separated from heparinized blood by density gradient centrifugation using Lymphocyte Separation Solution (Sangon Biotech, China). Total genomic DNA was extracted from mononuclear cells using an Axyprep Multisource Genomic DNA Miniprep Kit (Axygen, America) based on the manufacturer’s instructions. We used mtDNA standards to draw the standard curve, and the extraction method of the mtDNA standard was as follows: total DNA that was used for constructing plasmid DNA for human mtDNA was extracted from human platelets. Then, through steps such as PCR, transformation, distilling plasmid, connection and purification, plasmid DNA for human mtDNA was obtained.

### Measurement of mtDNA copy number

Our strategy determined the mononuclear cell mtDNA relative copy number as follows: 1) To obtain the mtDNA absolute value through mitochondrially encoded NADH: ubiquinone oxidoreductase core subunit 1 (MT-ND1) plasmid construction; and 2) to normalize against cell number using nuclear DNA (nDNA).

**Fig 1:**
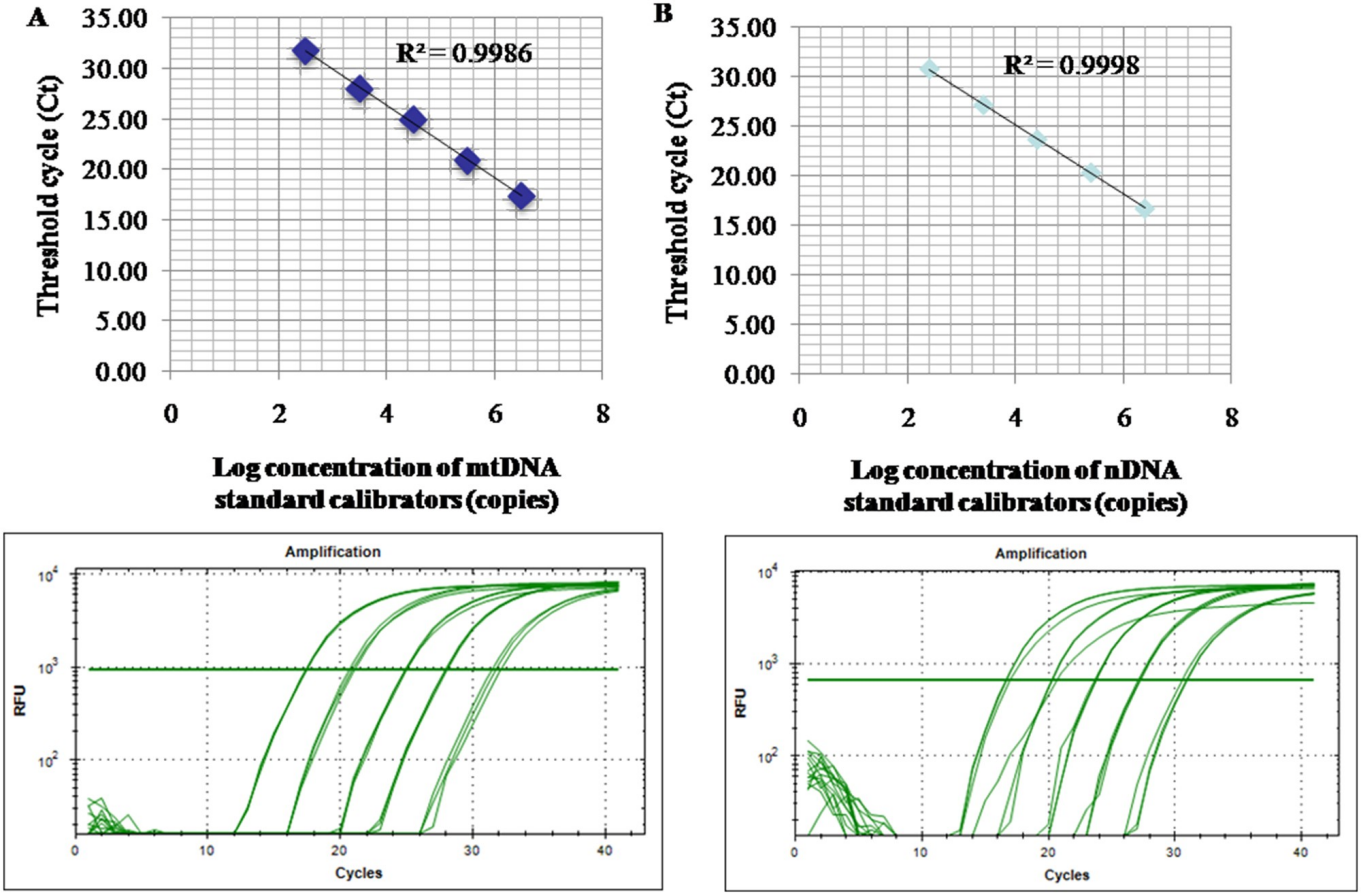
Representative standard curve and amplification plot from patient samples. (A) NADH dehydrogenase 1 complementary DNA was serially (1:10) diluted to prepare a series of standards (mtDNA standard) with known DNA copy number. The assay was linear over the range (325.195–3251950 copies) of DNA copy numbers (R^2^ = 0.9986). The amplification plot shows no irregular amplification for the standard diluents. (B) Beta ACTIN complementary DNA was serially (1:10) diluted to prepare a series of calibrators (nDNA standard) with known DNA copy number. The assay was linear over the range (255.487–2554870 copies) of DNA copy numbers (R^2^ = 0.9998). The amplification plot shows no irregular amplification for the standard diluents.

The relative amount of mtDNA per cell was measured using quantitative PCR and was normalized against the amount of nDNA. In short, for the determination of the amounts of mtDNA, we amplified a 108-bp mtDNA fragment in the MT-ND1 gene using primers 5’-ACA CTA GCA GAG ACC AAC CG-3’ and 5’-GAA GAA TAG GGC GAA GGG GC-3’, while for nDNA, a 90-bp fragment in the β-actin gene was amplified using 5’-TAA AGC GGC CTT GGA GTG TG-3’ and 5’-GAA CAC GGC TAA GTG TGC TG-3’. Each sample was measured in triplicate. Quantitative-PCR conditions were an initial denaturing step of 5 min at 95 °C followed by 40 cycles of 30 s denaturation at 95 °C, 30 s annealing at 55 °C, and 30 s extension at 72 °C. Every analysis included a negative control. A reference curve consisting of a serial dilution of a standard DNA was used for the quantification of mtDNA and nDNA, and the mtDNA content was calculated as the mtDNA/nDNA ratio in arbitrary units. A representative standard curve and amplification plot are shown in Fig 1.

### Statistical analysis

Data were expressed as the mean± SD or median (interquartile range). Univariate analyses were compared using independent two-sample Student’s t-test or analysis of variance while categorical variables were compared using χ2 test, as appropriate. The Cox proportional hazard survival model was applied to identify the independent contribution of prognostic factors to the prediction of the 28-day outcome. When constructing the multivariate model, univariate factors with *p*-values less than 0.2 were used. Hazard ratios with 95% confidence intervals (CIs) were used to estimate the associations between the independent variables and dependent variables. Survival curves were estimated using the Kaplan-Meier method. Statistical analysis was performed using SPSS Statistics 23 (IBM, New York). A two-sided *p* value of <0.05 was considered statistically significant.

### Ethics approval and consent to participate

The ethics committee of the First Affiliated Hospital, College of Medicine, Zhejiang University approved the protocol. Written informed consent was obtained from each patient.

## Results

We recruited 200 adult sepsis patients in the ICU. The baseline characteristics of survivors and nonsurvivors are presented in Table 1. The most common initial site of infection in both groups was the pulmonary system. Univariate analysis indicated that survivors were significantly different from nonsurvivors with respect tocertain demographic and clinical characteristics, including lower SOFA score, lower levels of white blood cells and blood urea nitrogen (BUN), and higher mononuclear cell mtDNA copy number (406.68 vs. 320.57, *p* = 0.001). Meanwhile, age, gender, underlying disease, site of initial suspected infection, ICU length of stay, mean platelet number, hemoglobin, lactate, aspartate transaminase (AST), alanine aminotransferase (ALT), serum creatinine (SCr), and C-reactive protein (CRP) demonstrated no significant difference between survivors and nonsurvivors.

**Table 1:** Baseline characteristics in septic patients.

Variable	Survivors (n = 138)	Nonsurvivors (n = 62)	P value
**Age (y)**	59.70±15.05	61.60±18.08	0.471
**Male (n, %)**	100 (72.46)	49 (79.03)	0.324
**Underlying diseases**			
Diabetes mellitus (n, %)	19 (13.77)	15 (24.19)	0.069
Hypertension (n, %)	46 (33.33)	27 (43.55)	0.165
Cardiac disease (n, %)	21 (15.22)	12 (19.35)	0.466
Nephropathy (n, %)	5 (3.62)	3 (4.84)	0.685
**Initial suspected infection site (n, %)**			0.950
Lung	99 (71.74)	44 (70.97)	
Abdomen	20 (14.49)	10 (16.13)	
Other	19 (13.77)	8 (12.90)	
**ICU length of stay (days)**	6.00 (4.00–13.00)	8.00 (3.00–15.00)	0.287
**SOFA score**	6.08±2.50	7.81±3.62	0.001
**Laboratory data**†			
WBC (×10^9^ /L)	12.20±6.05	16.29±8.85	0.001
Platelet (×10^9^ /L)	189.32±112.10	172.84±80.58	0.299
Hemoglobin (g/L)	109.83±28.53	106.97±25.53	0.499
Lactate (mmol/L)	2.42±2.36	2.71±2.09	0.408
AST (IU/L)	30.00 (20.00–72.00)	48.00 (33.00–153.00)	0.154
ALT (IU/L)	23.00 (12.00–71.00)	36.00 (24.50–79.00)	0.173
SCr (μmol/L)	104.41±100.56	116.97±74.66	0.380
BUN (mmol/L)	9.07±6.88	13.98±8.96	<0.001
CRP (mg/L)	71.64±67.77	75.70±69.37	0.698
**Mononuclear cell mtDNA relative copy number**	406.68 (196.65–625.35)	320.57 (175.98–437.33)	0.001

Note: ICU, intensive care medicine; SOFA, sequential organ failure assessment; WBC, white blood cell; AST, aspartate transaminase; ALT, alanine aminotransferase; SCr, serum creatinine; BUN, blood urea nitrogen; CRP, C-reactive protein;

^†^, laboratory data at presentation in the patients group.

**Fig 2:**
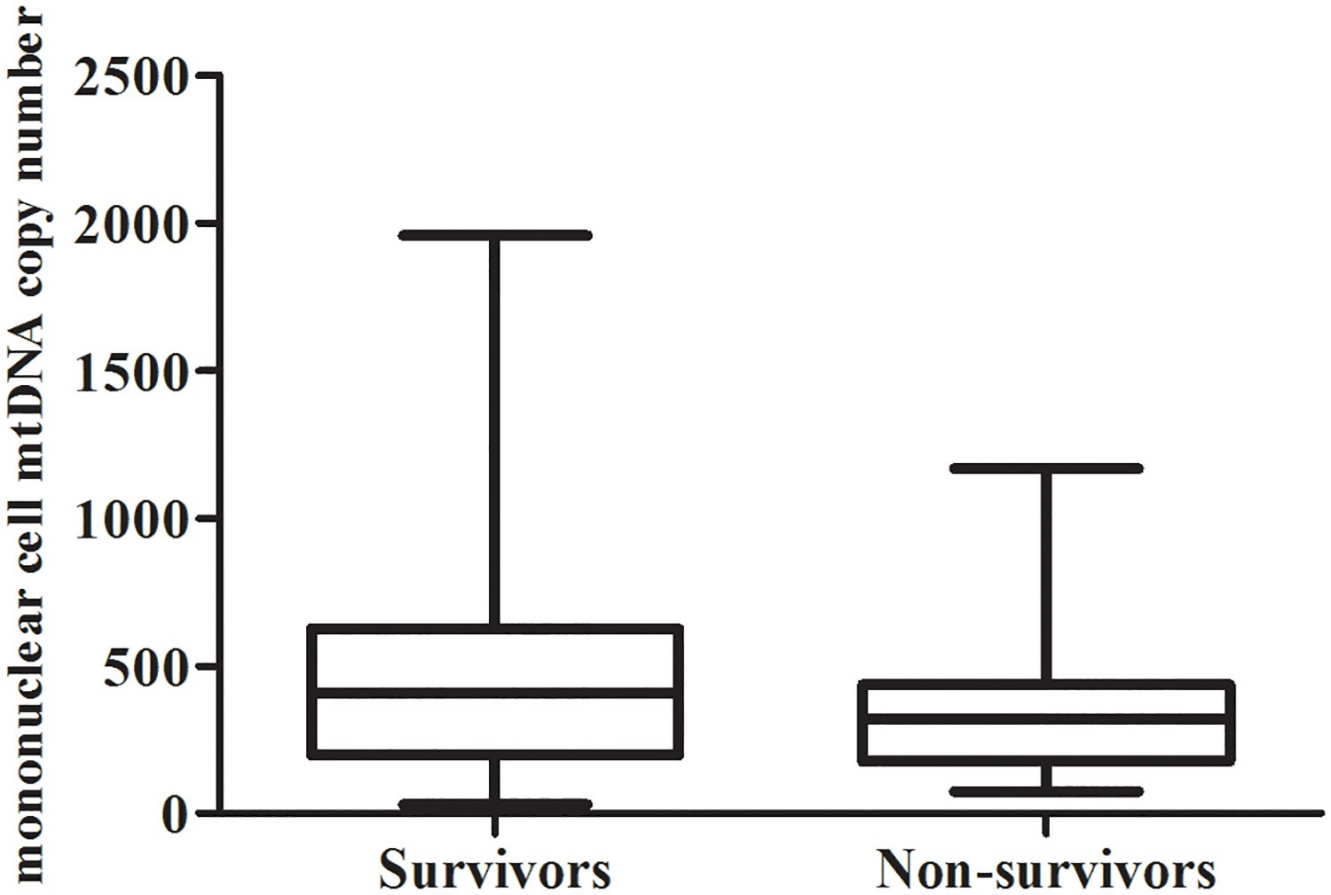
Box plots of the relative amount of mononuclear cell mtDNA copy number between survivors and nonsurvivors. The survivors had median mononuclear cell mtDNA copy number of 406.68. The median mononuclear cell mtDNA copy number for nonsurvivors was significantly lower at 320.57 (*p* = 0.001).

Mononuclear cell relative mtDNA copy number in sepsis patients between survivors and nonsurvivors is shown in Fig 2. The median mononuclear cell relative mtDNA copy number of survivors was significantly higher than that of nonsurvivors (406.68, range 196.65–625.35 vs. 320.57, range 175.98–437.33, *p* = 0.001).

We selected the univariate factors with p-values less than 0.2 (Table 1), including SOFA score, diabetes mellitus, hypertension, blood levels of white blood cells, AST, ALT and BUN, and mononuclear cell relative mtDNA copy number, and included them in the Cox proportional hazard survival model. We found that mononuclear cell relative mtDNA copy number was significantly negative associated with the 28-day mortality. For every additional unit of mononuclear cell mtDNA relative copy number, the risk of death falls by 0.1% (HR = 0.999, 95% CI = 0.998 to 1.000, *p* = 0.017, Table 2).

**Table 2:** Cox regression for 28-day mortality in sepsis patients.

Variable	HR (*95% CI*)	P value
**SOFA score**	1.085 (1.011–1.164)	0.023
**Underlying disease**		
Diabetes mellitus (%)	0.765 (0.412–1.420)	0.396
Hypertension (%)	0.619 (0.359–1.069)	0.086
**Laboratory data**†		
WBC (×10^9^ /L)	1.050 (1.019–1.083)	0.001
AST (IU/L)	1.000 (1.000–1.000)	0.068
ALT (IU/L)	1.000 (1.000–1.000)	0.817
BUN (mmol/L)	1.046 (1.021–1.072)	<0.001
**Mononuclear cell mtDNA relative copy number**	0.999 (0.998–1.000)	0.017

Note: HR, Hazard ratio; CI, confidence interval; SOFA, sequential organ failure assessment; WBC, white blood cell; AST, aspartate transaminase; ALT, alanine aminotransferase; BUN, blood urea nitrogen;

^†^, laboratory data at presentation in the patients group.

**Fig 3:**
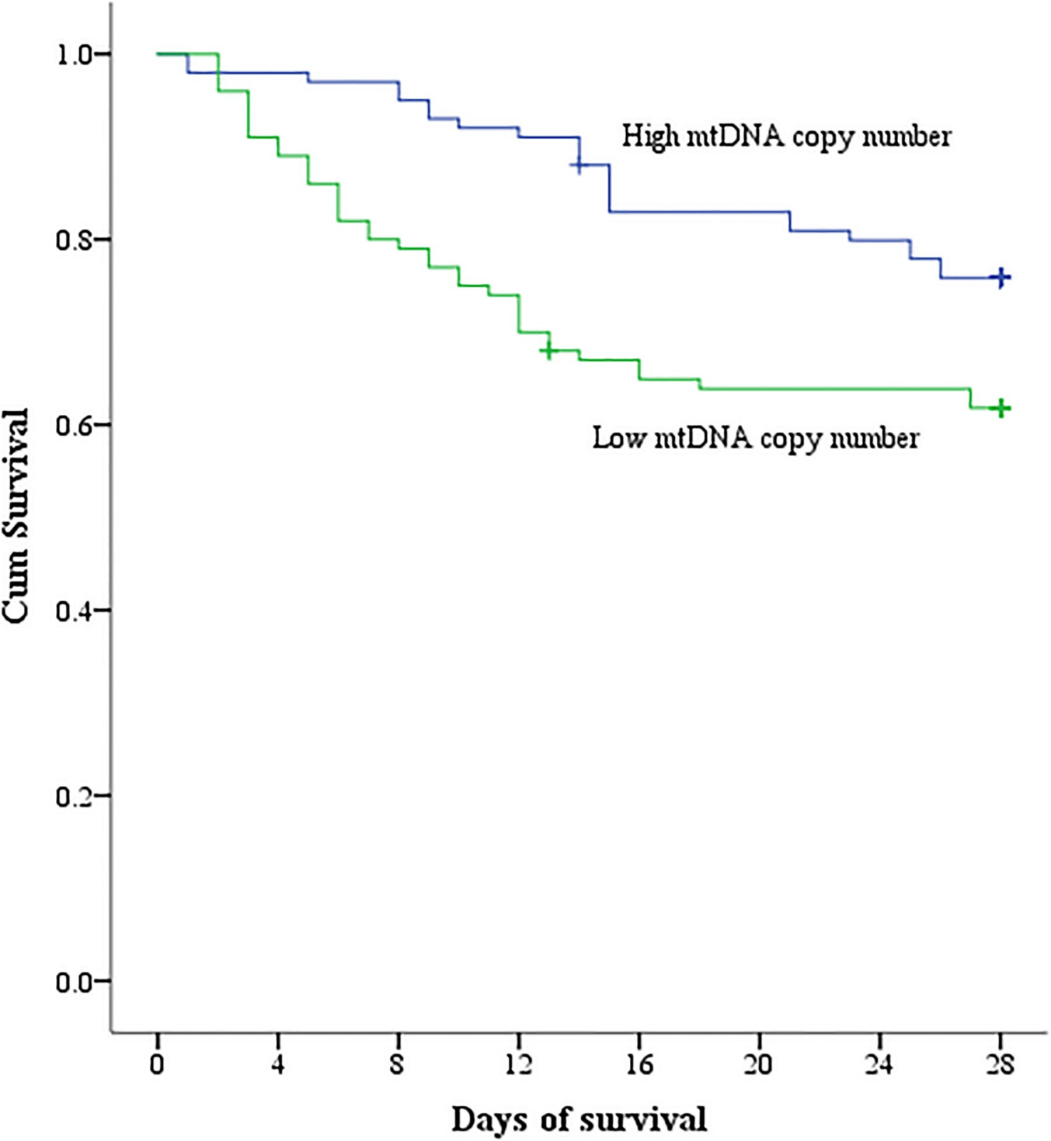
Kaplan–Meier analysis. Compared with high mononuclear mtDNA copy number, those with low mtDNA copy number had a higher rate of 28-day mortality (*p*<0.05).

Then we divided the total recruited patients into following two groups to perform subgroup analysis: high mtDNA copy number group was defined as patient median mononuclear mtDNA copy number ≥ 362.61, and < 362.61 defined the low group. Kaplan-Meier analysis indicated significantly higher survival over 28 days in patients with high mononuclear mtDNA copy number than in those with low mtDNA copy number (*p*<0.05, Fig 3).

The baseline demographics and clinical characteristics of high mtDNA copy number group and low group were summarized in Table 3. Compared with the high mononuclear cell mtDNA copy number group, the low group had higher mortality.

**Table 3:** Baseline characteristics of the high mononuclear cell mtDNA copy number group and the low group in sepsis patients.

Variable	High mtDNA copy number (≥362.61, n = 100)	Low mtDNA copy number (<362.61, n = 100)	P value
**Age (y)**	60.55±15.74	60.02±16.38	0.816
**Male (n, %)**	75 (75.00)	74 (74.00)	0.871
**Underlying diseases**			
Diabetes mellitus (n, %)	14 (14.00)	20 (20.00)	0.259
Hypertension (n, %)	40 (40.00)	33 (33.00)	0.304
Cardiac disease (n, %)	18 (18.00)	15 (15.00)	0.568
Nephropathy (n, %)	2 (2.00)	6 (6.00)	0.149
**Initial suspected infection site (n, %)**			0.546
Lung	68 (68.00)	75 (75.00)	
Abdomen	17 (17.00)	13 (13.00)	
Other	15 (15.00)	12 (12.00)	
**ICU length of stay (days)**	6.00 (4.00–13.00)	6.00 (3.00–15.75)	0.649
**SOFA score**	6.44±2.82	6.79±3.16	0.410
**In-hospital mortality (n, %)**	24 (24.00)	38 (38.00)	0.032
**Laboratory data**†			
WBC (×10^9^ /L)	13.79±7.15	13.14±7.40	0.527
Platelet (×10^9^ /L)	174.09±94.28	194.33±111.42	0.167
Hemoglobin (g/L)	109.03±29.03	108.86±26.24	0.965
Lactate (mmol/L)	2.43±2.24	2.59±2.32	0.625
AST (IU/L)	35.00 (22.00–72.00)	38.00 (23.00–104.00)	0.137
ALT (IU/L)	26.00 (12.00–68.00)	30.00 (14.00–79.00)	0.952
SCr (umol/L)	121.27±108.23	95.34±73.79	0.049
BUN (mmol/L)	10.85±8.67	10.33±7.10	0.644
CRP (mg/L)	73.39±68.70	72.41±67.88	0.919

Note: ICU, intensive care medicine; SOFA, sequential organ failure assessment; WBC, white blood cell; AST, aspartate transaminase; ALT, alanine aminotransferase; SCr, serum creatinine; BUN, blood urea nitrogen; CRP, C-reactive protein

^†^, laboratory data at presentation in the patients group.

## Discussion

The definition of a disease is the cornerstone for the consistency among epidemiologic studies and clinical trialsand facilitates earlier recognition and more timely management for patients suffering from the disease. The definitions of sepsis have remained largely unchanged for more than 2 decades [15]: the diagnosis of sepsis required suspected or confirmed infection and the presence of 2 or more systemic inflammatory response syndrome criteria. The previous definition was broad, and some controversy regarding investigation might have been due to the previous definition itself, in particular in clinical trials. The updated definition of sepsis has been published consistent with the improved understanding of sepsis pathobiology [2]. Therefore, it is necessary to re-evaluate the data based on previous sepsis definition, especially those generating controversy, even if only to replicate the research. It is widely believed that mitochondria play important roles in the pathogenesis of infection-induced diseases [16, 17]. Although mitochondria associated mechanisms are not completely clear in sepsis, to date, it was proved that the scenarios of mitochondrial effect on disease were quite different between clinical settings with or without organ dysfunction [7]. The most obvious alteration of the updated definition of sepsis was reflected in the new criterion of a SOFA score of no less than 2. According to the Sepsis-3 definition, all diagnoses of sepsis must include clear organ injury secondary to infection. Therefore, the updated definition is important for studies concerning mitochondrial effect on sepsis. In the present study, we analyzed mononuclear cell mtDNA copy number in samples of sepsis patients according to the Sepsis-3 definition. Our data indicated that the relative amount of mononuclear cell mtDNA copy number in nonsurvivors was significantly lower than that of survivors. Moreover, mononuclear cell mtDNA copy number was an important predictor of clinical outcome, demonstrating that patients with low copy number had high 28-day mortality. A previous study found that mtDNA depletion occurred in mononuclear cells of sepsis patients and correlated with disease severity [18]. Obviously, the results of the present study agreed with those of the previous one. SOFA scores were not found in the previous study; however, it was clear that patients diagnosed as having severe sepsis, according to European/North American consensus criteria, were recruited in the study cohort. The consistency between the results of the two studies might imply that the content of the updated definition of sepsis was equivalent to the definition of severe sepsis, according to European/North American consensus criteria [19]. Any potential evidence illuminating the relationship between the two definitions of sepsis would help investigators to accurately and comprehensively understand the conclusions drawn from various studies associated with sepsis.

As described above, numerous lines of evidence found a depletion of mitochondrial copy number associated with the susceptibility and pathogenesis of sepsis-associated organ injury [20, 21]. Sepsis activates several pathological mechanisms linked to mitochondria, including hypoperfusion, oxidative stress, and the inflammatory response. Disturbance of those pathways produces abundant reactive oxygen species (ROS) and decrease antioxidant defenses, disrupting mitochondrial integrity [7]. As mtDNA is structurally close to the inner mitochondrial membrane that produces ROS and lacks sufficient repair mechanisms, it is extremely vulnerable to damage. Damaged mtDNA could be eliminated by autophagy, resulting in loss in the number of mtDNA copy [22].

Mononuclear cells have the function of regulating the innate immune response to infection [23]. Monocytes and lymphocytes play important roles in the immune process, including antigen processing, presentation and amplification of response. Changing mtDNA copy number has been considered to be indispensable in cell proliferation and differentiation; for example, activation of T cell requires a rapid increase in mtDNA copy number [24]. Therefore, it is presumable that low mtDNA copy number in mononuclear cells will damage the immune response, which is known to be crucial for recovery from sepsis [25].

Sepsis is a severe threat to human health. Early and accurate risk stratification of sepsis will facilitate timely adjustment of the therapeutic strategies for these patients. The increased variety of biomarkers and the improvement of the scoring system provide new insights into the diagnosis and prognosis of sepsis. Nevertheless, new outcome risk factors or predictors remain needed to evaluated severty of sepsis, if only to incorporate them into the existing scoring system, for the sake of improving the accuracy of evaluating severity in sepsis. According to the present study, circulating mononuclear cellular mtDNA copy number might be helpful for outcome prediction in sepsis; low mtDNA copy numbers implied poor prognosis.

The present study had limitations. We did not perform a dynamic view of the circulating mononuclear cell mtDNA copy number variants during the 28-day period. The study might have limited value to evaluate organ dysfunction based on mononuclear cells. Furthermore, this study only contributed to exploring associations; not causation. Therefore, further study is required to precisely evaluate the relationship between mononuclear cellular mtDNA copy number and prognosis of sepsis patients.

## Conclusions

Circulating mononuclear cell mtDNA copy number was negatively associated with 28-day mortality in sepsis patients. For every additional unit of mononuclear cell mtDNA relative copy number, the risk of death falls by 0.1% (HR = 0.999, 95% CI = 0.998 to 1.000, *p* = 0.017). Our data indicated that circulating mononuclear cellular mtDNA copy number might be helpful for the outcome prediction in sepsis patients, with lower mtDNA copy number implying poor prognosis.

## Supporting information

S1 File Raw clinical data of septic patients in this study. (XLSX)

S1 Checklist STROBE_checklist. (DOCX)
